# Thyroid nodule recognition in computed tomography using first order statistics

**DOI:** 10.1186/s12938-017-0367-2

**Published:** 2017-06-02

**Authors:** Wenxian Peng, Chenbin Liu, Shunren Xia, Dangdang Shao, Yihong Chen, Rui Liu, Zhiping Zhang

**Affiliations:** 1 Key Laboratory of Biomedical Engineering of Ministry of Education, Zhejiang University, Hangzhou, Zhejiang China; 2 Zhejiang Provincial Key Laboratory of Cardio-Cerebral Vascular Detection Technology and Medicinal Effectiveness Appraisal, Zhejiang University, Hangzhou, Zhejiang China; 3 Radiology Department, Hangzhou Medical College, Hangzhou, Zhejiang China; 4 Biodesign Institute, Arizona States University, Tempe, AZ USA; 5 Radiology Department, Tongde Hospital of Zhejiang Province, Hangzhou, Zhejiang China

**Keywords:** Thyroid nodule, Computed tomography, Texture feature, Texture analysis

## Abstract

**Background:**

Computed tomography (CT) is one of the popular tools for early detection of thyroid nodule. The pixel intensity of thyroid in CT image is very important information to distinguish nodule from normal thyroid tissue. The pixel intensity in normal thyroid tissues is homogeneous and smooth. In the benign or malignant nodules, the pixel intensity is heterogeneous. Several studies have shown that the first order features in ultrasound image can be used as imaging biomarkers in nodule recognition.

**Methods:**

In this paper, we investigate the feasibility of utilizing the first order texture features to identify nodule from normal thyroid tissue in CT image. A total of 284 thyroid CT images from 113 patients were collected in this study. We used 150 healthy controlled thyroid CT images from 55 patients and 134 nodule images (50 malignant and 84 benign nodules) from 58 patients who have undergone thyroid surgery. The final diagnosis was confirmed by histopathological examinations. In the presented method, first, regions of interest (ROIs) from axial non-enhancement CT images were delineated manually by a radiologist. Second, average, median, and wiener filter were applied to reduce photon noise before feature extraction. The first-order texture features, including entropy, uniformity, average intensity, standard deviation, kurtosis and skewness were calculated from each ROI. Third, support vector machine analysis was applied for classification. Several statistical values were calculated to evaluate the performance of the presented method, which includes accuracy, sensitivity, specificity, positive predictive value (PPV), negative predictive value (NPV), and area of under receiver operating characteristic curve (AUC).

**Results:**

The entropy, uniformity, mean intensity, standard deviation, skewness (P < 0.05), except kurtosis (P = 0.104) of thyroid tissue with nodules have a significant difference from those of normal thyroid tissue. The optimal classification was obtained from the presented method. The accuracy, sensitivity, specificity, positive predictive value (PPV) and negative predictive value (NPV) are 0.880, 0.821, 0.933, 0.917, 0.854, and 0.953 respectively.

**Conclusion:**

First order texture features can be used as imaging biomarkers, and the presented system can be used to assist radiologists to recognize the nodules in CT image.

## Background

According to the National Cancer Institute’s Surveillance, Epidemiology, and End Results (SEER) program, the number of new cases of thyroid cancer has been increased from 4.85 to 15.07 per 100,000 men and women since 1975. The incidence rate is about 98.2 per 100,000 among people aged 35–54 [1]. A larger number of mid-age patients cost the whole nation a lot for diagnosis, surgery, and adjuvant therapy. Thyroid nodules are very common: the prevalence of palpable nodules is about 4 ~ 8%. The prevalence of thyroid nodules identified by means of pathologic examination at autopsy approaches 50% [2, 3]. Although thyroid cancer accounts for only a small proportion of thyroid nodules, about 5% [4], an accurate and efficient diagnostic tool is critical for patients to detect thyroid nodules.

The important and first step of the successful treatment is that nodules could be diagnosed at an early stage. With the development of imaging technology and image processing, thyroid nodule diagnosis becomes an increasingly frequent event. Currently, the widely used imaging methods for thyroid nodules include ultrasound, magnetic resonance imaging (MRI), computed tomography (CT), and positron emission tomography (PET) [5–7]. Ultrasound is a key diagnostic tool in the initial evaluation of thyroid nodules because it is low cost and convenient. The computer aided detection systems based on US images have been developed to help doctors identify nodule from normal thyroid tissues [8]. MRI has an adjuvant role in the evaluation of thyroid disease, and the utility of PET is in the evaluation of thyroid cancers with dedifferentiated tumours [8]. CT provides valuable information for further operative intervention, especially for retrosternal goiters, the malignant case with suspicion of extracapsular extension [9, 10], and multiple punctate calcifications [11]. The usage of CT scans helps in the detection of incidental thyroid cancers [12]. In clinical practice, radiologists visually inspect a large amount of CT images, which is a tedious and error-prone task. The reporting practices for incidental thyroid nodules (ITNs) are highly variable. Based on radiologist’s experience, practice type, and training [13]. Some subtle CT features, like calcification, could be missed in visual inspection. To overcome the limitations, computer aided detection (CAD) systems can be developed to improve the accuracy of radiologists in the interpretation of CT images.

Nowadays, there have been studies to assess the feasibility of CT images in thyroid nodule evaluation. Li assessed the thyroid nodules in dual-energy computed tomography imaging, and found a significant difference between benign and malignant groups in iodine concentration, Hounsfield unit (HU) curve slope, and effective atomic number [14]. Using a larger dataset, CT scans in 734 patients, Yoon found that rim calcifications, high anteroposterior-transverse diameter ratio and mean attenuation value suggest malignancy of the incidental thyroid nodules [15]. Several groups have attempted to predict malignancy from multiple punctate calcifications and solitary coarse calcification [11, 16]. Previous studies showed that the imaging characteristics of thyroid nodules in CT have promising potential for differentiation of benign and malignant thyroid nodules. However, there are no studies about CAD system to assess the imaging characteristics of thyroid in CT for nodule detection.

In this paper, we presented a CAD system to detect thyroid nodules in CT images. Six image features, including entropy, uniformity, mean intensity, standard deviation, and kurtosis, were extracted from thyroid regions. Three de-noising filters, including average, median, and wiener filter, were used and their effect on the performance of CAD system was evaluated. We further consider feature selection method to find the optimized feature subset and improve the classification accuracy. Here we report a light-weighted CAD system for thyroid nodules detection in CT images. This system has potential to lighten the radiologists’ burden and improve the diagnostic accuracy of thyroid nodules.

We arrange the present paper in the following orders. First, the inclusion criteria and vital parameters of our data were described. Second, the thyroid regions were delineated in CT images by an experienced radiologist. Third, texture features were extracted from the delineated regions, and support vector machine was applied to train and predict the nodules and normal tissues. Finally, we evaluate the performance of different feature subsets to improve the accuracy of the presented method.

## Methods

### Study population

From January 2013 to January 2014, thyroid images were found in 434 cases through non-enhanced CT examination of neck or chest in the picture archiving and communication system (PACS) of Ruian People Hospital, Zhejiang, China. Nodule cases without surgical treatment and pathological result (n = 301) and cases with inappropriate CT protocol, poor image quality (n = 20) were excluded. Finally, 58 nodule cases with surgical treatments and pathological results and 55 health controlled cases (mean age 52.0 ± 13.5 years; range 25–80 years) met the inclusion criteria. Two or three images were selected from each case (Table 1).

**Table 1:** Patient and image information in this study

	Benign			Malignant		Normal	Total
	Goiters	Thyroiditis	Thyroid Adenoma	PTC	FTC		
Patients	30	3	4	20	1	55	113
Images	63	9	12	47	3	150	284

*PTC* papillary thyroid cancer, *FTC* follicular thyroid cancer

### CT examinations

The scanning was conducted with 16-channel Helical CT scanner (Sensation, Siemens Medical Solution, Erlangen, Germany). The patients lay in supine position and were scanned from pharynx oralis to the upper edge of the clavicle, and some were scanned to tracheal bifurcation. The scanning parameters were: 120 kVp, with CARE DOSE 4D technology, 0.6 mm × 16 of collimation, 1 of pitch, 0.5 s of frame rotation, 2 ~ 3 mm of slice thickness and same cross-sectional distance, B31 standard of reconstruction kernel.

### Regions of interest

**Fig. 1:**
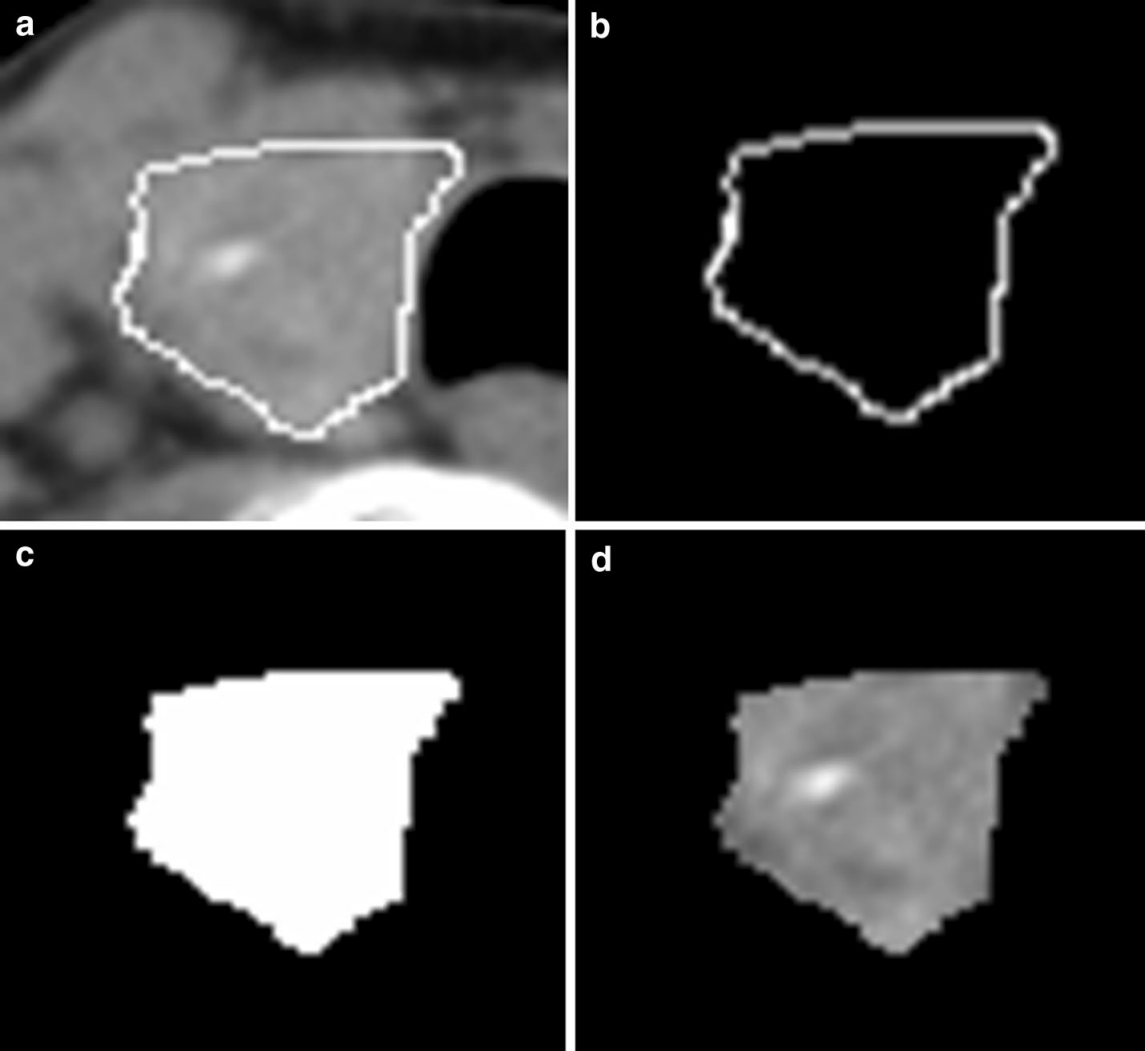
The procedure of ROI extraction in thyroid CT image. **a** The contour of a thyroid with malignant nodule. **b** Binary contour. **c** Mask of thyroid tissue. **d** Target ROI of thyroid tissue

To make sure the image quality, CT images were checked in PACS station (Maroland iEIS, m-Viewer version 5.3, China) by an experienced radiologist. One to three regions of interest (ROIs) in transverse non-enhancement CT images were selected from each case. The contours of thyroid tissues on each image were delineated manually by the experienced radiologist with MRIcro software (MRIcro by Chris Rorden, version 1.39 build 5). Finally, ROIs (nodule, n = 134; normal, n = 150) were extracted. The main steps of segmentation include: (1) The contour of single thyroid (Fig. 1a). (2) The A was converted into binary image (Fig. 1b). (3) The region was filled with number one and saved as a mask (DICOM format) (Fig. 1c). (4) The target image was obtained by multiplying the original image with the mask (Fig. 1d). Our calculation platform was Matlab R2012b (8.0.0.783), windows XP.

### Feature extraction and normalization

The normal thyroid tissue is homogeneous in image intensity. However, for thyroid nodules, spatial heterogeneity is a well-recognized feature that reflects the area of necrosis, haemorrhage, and calcifications [17]. The quantification of heterogeneity can be used as an imaging biomarker to differentiate between tumour types, grade tumours, and predict outcome [18]. In our study, we used first order texture features as the quantification of heterogeneity, including entropy (irregularity), uniformity (distribution of gray level), mean intensity (intensity level), kurtosis (magnitude of intensity distribution), skewness (skewness of intensity distribution), and standard deviation. The texture feature equations are listed in Table 2. The photon noises can cause heterogeneity in CT imaging, which may mask the underlying biological heterogeneity. To reduce these noises, three filters, including average, median and wiener filters, were used as the image pre-processing. The window size of the filter is 3 * 3 pixels. The first order texture features were calculated both with and without filters. In general, high entropy, standard deviation, and kurtosis, and low uniformity and skewness indicate heterogeneous tissues, which could be nodules.

**Table 2:** Descriptions and equations of first-order texture features used in this study

Feature type	Equations	Description
Entropy	$e = - \mathop \sum \limits_{{l = 1}}^{k} \left[ {p\left( l \right)} \right]\log _{2} \left[ {p\left( l \right)} \right]$	Describes the randomness and irregularity of all pixel intensity
Uniformity	$u = \mathop \sum \limits_{{l = 1}}^{k} \left[ {p\left( l \right)} \right]^{2}$	Describes the distribution of gray level degree
Mean intensity	$m = \frac{1}{\text{n}}\mathop \sum \limits_{{{\text{i}} = 1}}^{\text{n}} p(i)$	Describes the mean intensity value of all pixels
Standard deviation	$sd = \left( {\frac{1}{{\left( {n - 1} \right)}}\mathop \sum \limits_{{\left( {x,y} \right) \in R}} \left[ {{\text{a}}\left( {x,y} \right) - \overline{\text{a}} } \right]^{2} } \right)^{{\frac{1}{2}}}$	Describes the off variation from the mean pixel value
Kurtosis	$$\begin{aligned} k &= \frac{{n\left( {n + 1} \right)}}{{\left( {n - 1} \right)\left( {n - 2} \right)\left( {n - 3} \right)}}\frac{{\mathop \sum \nolimits_{{\left( {x,y} \right) \in R}} \left[ {{\text{a}}\left( {x,y} \right) - \overline{\text{a}} } \right]^{4} }}{{\left[ {sd\left( a \right)} \right]^{4} }} \\ & \quad - 3\frac{{\left( {n - 1} \right)^{2} }}{{\left( {n - 2} \right)\left( {n - 3} \right)}} \end{aligned}$$	Indicates the bulging or peakedness
Skewness	$s = \frac{n}{{\left( {n - 1} \right)\left( {n - 2} \right)}}\frac{{\mathop \sum \nolimits_{{\left( {x,y} \right) \in R}} \left[ {{\text{a}}\left( {x,y} \right) - \overline{\text{a}} } \right]^{3} }}{{sd\left( a \right)^{3} }}$	Indicates the asymmetry

All the features were normalized to [0, 1] according to Eq. (1):

1 $$ {{{\text{Y}}_{\text{i}} = \left( {{\text{X}}_{\text{i}} - { \hbox{min} }({\text{X}}_{\text{i}} )} \right)} \mathord{\left/ {\vphantom {{{\text{Y}}_{\text{i}} = \left( {{\text{X}}_{\text{i}} - { \hbox{min} }({\text{X}}_{\text{i}} )} \right)} {\left( {\hbox{max} \left( {{\text{X}}_{\text{i}} } \right) - \hbox{min} \left( {{\text{X}}_{\text{i}} } \right)} \right)}}} \right. \kern-0pt} {\left( {\hbox{max} \left( {{\text{X}}_{\text{i}} } \right) - \hbox{min} \left( {{\text{X}}_{\text{i}} } \right)} \right)}} $$

where X_i_ is the ith original feature, Y_i_ is the ith normalized feature.

### Statistics analysis

The normalized texture features are evaluated and compared between nodule and normal groups using independent-samples student’s *T* test. If the P value is less than 0.05, it indicates the difference of the feature between two groups is statistically significant. Receiver operating characteristic (ROC) curve was performed to illustrate the performance of the classifier system. The area under the receiver operating characteristic curve (AUC) was calculated to evaluate the accuracy of the classification.

### Feature selection

To remove the redundant features and improve the performance of classification, we used sequential forward floating selection (SFFS) to select optimized feature subset [19]. The criterion used to select features was the accuracy of the k-nearest neighbour classification. The method started from an empty feature set, and created candidate feature subsets by sequentially adding each of the features not yet selected. For each candidate feature subset, leave-one-out cross validation was used. The selected feature subset was the one that had optimal classification performance. To validate the selected feature subset, we randomly divided all the samples into two groups, selection group and validation group. We used samples in the selection group to find the optimal feature subset with SFFS method. The selected feature subset was validated with samples in the validation group.

### Classification

Computer-aided diagnosis/detection often implies processing large scale and high dimensional datasets [20, 21]. Recent studies on local binary pattern and deep learning can extract high-level contents in images and achieve efficient recognition in several large datasets [22, 23]. As a preliminary study, our dataset is small, so we focus on the feasibility of first order texture features to identify nodule from normal thyroid tissue. Support Vector Machine (SVM) is a classic pattern recognition method introduced by Vapnik since 1995, which is successfully used in solving a range of problems, especially in the case of small scale samples, high-dimensional data, and non-linear pattern recognition [18, 24, 25]. We used SVM in this study to classify the nodule from the normal tissues.

If given a training sample set $\left\{ {\left( {{\text{x}}_{\text{i}} , {\text{ y}}_{\text{i}} } \right)} \right\}_{\text{i = 1}}^{{^{\text{n}} }}$, where x_i_ denotes the training vector, x_i_∊R^n^ and y_i_ denotes the corresponding class label, the value of y_i_ is 1 or −1, and n denotes the total number of the training sample. SVM will find the solution of the following optimization problem:

2 $$ \min_{{{\text{w}},{\text{b}},\xi }} \frac{1}{2}\left\langle {{\text{w}}^{T} \cdot {\text{w}}} \right\rangle + {\text{C}}\mathop \sum \limits_{{{\text{i}} = 1}}^{\text{n}} \xi_{\text{i}} $$

$$\text{Subject\;to}{:}\;{\text{y}}_{\text{i}} \left( {\left\langle {{\text{w}} \cdot {\text{x}}_{\text{i}} } \right\rangle + {\text{b}}} \right)  + \xi_{\text{i}} - 1 \; \ge \;0 $$

Here C is a penalty parameter of the error term, *ξ*_i_ is the non-negative slack variable, w is the normal vector of the hyper-plane, and b is the offset of the plane. SVM will find the linear separating hyper-plane with the maximal marginal in higher dimensional space. Then, a kernel function ${\text{K}}({\text{x}}_{{\text{i}}} {\text{,x}}_{{\text{j}}} ) = {{\upvarphi }}({\text{x}}_{{\text{i}}} )^{{\text{T}}} {{\upvarphi }}({\text{x}}_{{\text{j}}} ){\text{i}}$ is used to map the training sample into a higher dimensional feature space. In our study, the SVM parameters were optimized by grid search using cross-validation, and the radial basis function (RBF) was used as the kernel of SVM.

To assess the performance of the presented methods, six objective indices, including sensitivity (SEN), specificity (SPC), accuracy (ACC), positive predictive value (PPV) and negative predictive value (NPV), were calculated.

These indices are defined as follows:

3 $$ {\text{Sensitivity}}\left( {\text{SEN}} \right) = {{{\text{N}}_{\text{TP}} } \mathord{\left/ {\vphantom {{{\text{N}}_{\text{TP}} } {\left( {{\text{N}}_{\text{TP}} + {\text{N}}_{\text{FN}} } \right)}}} \right. \kern-0pt} {\left( {{\text{N}}_{\text{TP}} + {\text{N}}_{\text{FN}} } \right)}} $$

4 $$ {\text{Specificity}}\left( {\text{SPC}} \right) = {{{\text{N}}_{\text{TN}} } \mathord{\left/ {\vphantom {{{\text{N}}_{\text{TN}} } {\left( {{\text{N}}_{\text{TN}} + {\text{N}}_{\text{FP}} } \right)}}} \right. \kern-0pt} {\left( {{\text{N}}_{\text{TN}} + {\text{N}}_{\text{FP}} } \right)}} $$

5 $$ {\text{Posittive Predictive Value}}\left( {\text{PPV}} \right) = {{{\text{N}}_{\text{TP}} } \mathord{\left/ {\vphantom {{{\text{N}}_{\text{TP}} } {\left( {{\text{N}}_{\text{TP}} + {\text{N}}_{\text{FP}} } \right)}}} \right. \kern-0pt} {\left( {{\text{N}}_{\text{TP}} + {\text{N}}_{\text{FP}} } \right)}} $$

6 $$ {\text{Negative Predictive Value}}\left( {\text{NPV}} \right) = {{{\text{N}}_{\text{TN}} } \mathord{\left/ {\vphantom {{{\text{N}}_{\text{TN}} } {\left( {{\text{N}}_{\text{TN}} + {\text{N}}_{\text{FN}} } \right)}}} \right. \kern-0pt} {\left( {{\text{N}}_{\text{TN}} + {\text{N}}_{\text{FN}} } \right)}} $$

7 $$ {\text{Accuracy}}\left( {\text{ACC}} \right) = {{\left( {{\text{N}}_{\text{TN}} + {\text{N}}_{\text{TP}} } \right)} \mathord{\left/ {\vphantom {{\left( {{\text{N}}_{\text{TN}} + {\text{N}}_{\text{TP}} } \right)} {\left( {{\text{N}}_{\text{TN}} + {\text{N}}_{\text{TP}} + {\text{N}}_{\text{FN}} + {\text{N}}_{\text{FP}} } \right)}}} \right. \kern-0pt} {\left( {{\text{N}}_{\text{TN}} + {\text{N}}_{\text{TP}} + {\text{N}}_{\text{FN}} + {\text{N}}_{\text{FP}} } \right)}} $$

where N_TP_ and N_TN_ are the numbers of nodule and normal cases respectively that were identified correctly. N_FP_ and N_FN_ are the numbers of nodule and normal cases respectively that were identified incorrectly.

## Results and discussion

### T test evaluation

Six texture features without filter were calculated and listed in Table 3. Entropy, uniformity, mean intensity, standard deviation and skewness have significant differences between nodule and normal groups in independent sample T test (all *P* value <0.05) except kurtosis (*P* value =0.104).

**Table 3:** The comparison of six features between nodule and normal thyroid tissue after normalization(mean ± SD)

Feature	Nodule	Normal	*P* value	*F* value
Entropy	0.828 ± 0.203	0.963 ± 0.020	<0.001	105.910
Uniformity	0.088 ± 0.175	0.007 ± 0.005	<0.001	76.834
Mean intensity	0.209 ± 0.098	0.257 ± 0.040	0.037	4.374
Standard deviation	0.677 ± 0.123	0.439 ± 0.218	0.001	11.286
Kurtosis	0.365 ± 0.102	0.317 ± 0.878	0.104	2.656
Skewness	0.547 ± 0.083	0.489 ± 0.052	0.002	10.190

The mean intensities of two groups without normalization are: 89.491 ± 17.295 HU, nodule: 68.851 ± 42.019 HU, P value <0.05

**Fig. 2:**
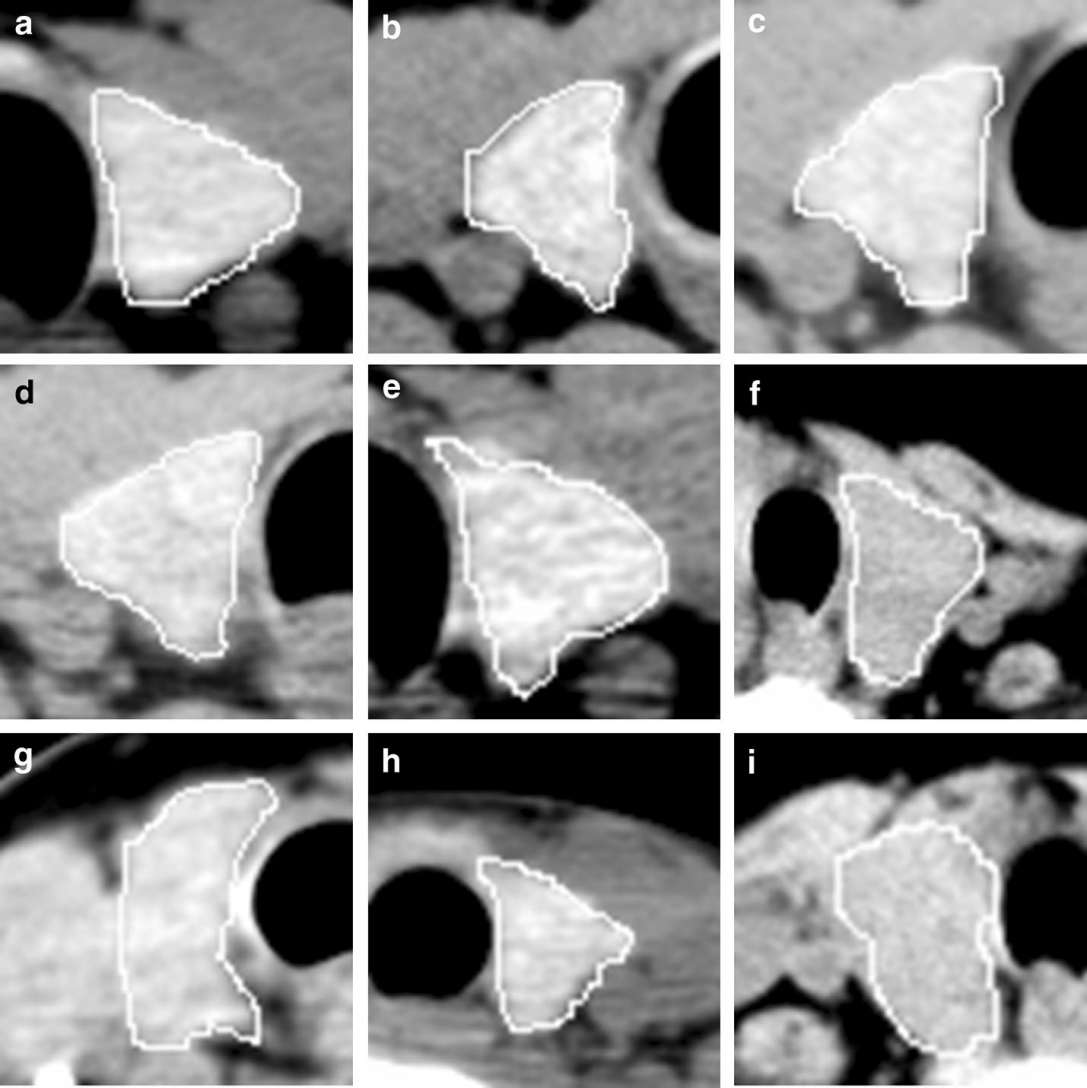
Thyroid ROIs in CT images. Images (**a**–**i**) are normal thyroid tissue from nine patients

**Fig. 3:**
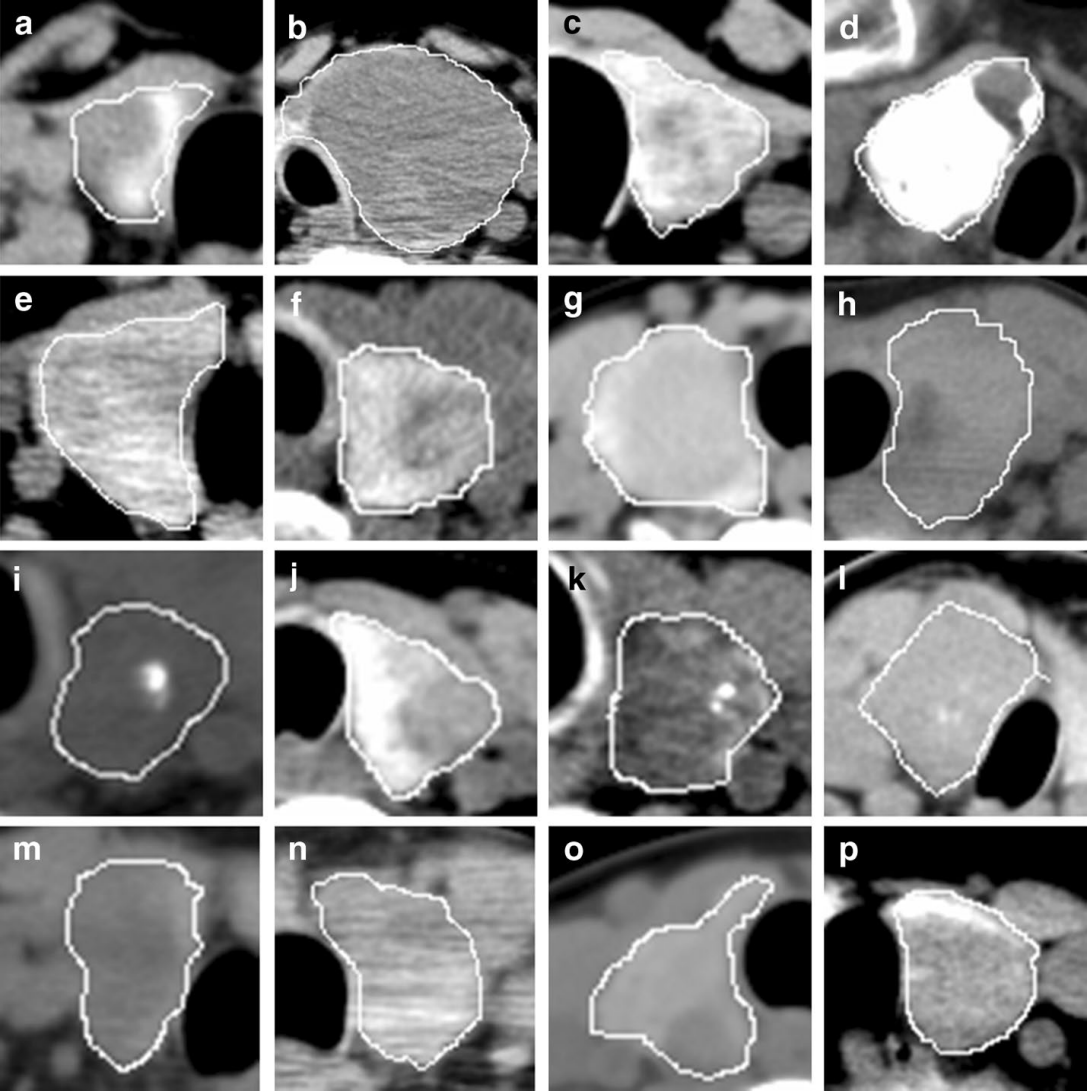
CT images of thyroid nodules from different patients with marked ROIs. Thyroid nodules in images (**a**–**h**) are benign. Nodules in images (**i**–**p**) are malignant

The pixel intensity in normal thyroid tissues (Fig. 2a–i) is homogeneous and smooth. In the benign (Fig. 3a–h) and malignant (Fig. 3i–p) nodules, the ROI intensity is heterogeneous. In the thyroid nodules, the tumour cell usually appears different from the normal thyroid cell. In general, normal thyroid tissue cell can absorb iodine. The average intensity (CT value) of the normal thyroid is around 90–120 HU. On the contrary, the tumour cell does not have the capability to absorb iodine as thyroid cell. The average intensity of the nodule is less than 70 HU, such as goiter, thyroiditis, and carcinoma. Besides, the intensity could be greatly more than 120 HU if calcification exists in thyroid gland. The intensity of the nodule in CT images varies due to different compositions. So the spatial heterogeneity in the thyroid tissue can be quantified with the first order statistics. And these statistics can be used as imaging biomarker to detect the thyroid nodules. In the following test, we also evaluated the performance of different filters, including average, median, and wiener filter.

### Classification results

To evaluate the performance of each feature without filter, we calculated the classification results with SVM classifier (Table 4). Entropy, uniformity, mean intensity, and skewness performed better than standard deviation and kurtosis. For standard deviation (AUC = 0.510) and kurtosis (AUC = 0.565), they have low sensitivity and high specificity (SEN < 0.100 and SPC > 0.900), which means almost all the samples were identified as negative ones. In this test, we can see the contribution of each single feature. However, it is impractical to use a single feature to characterize the thyroid image. Multiple features could improve the performance and make more robust decision. In the following section, we will evaluate the filters, introduce feature selection, and optimize the feature subsets.

**Table 4:** Classification results using each single feature

Feature	ACC	SEN	SPC	PPV	NPV	AUC
Entropy	0.792	0.612	0.953	0.921	0.733	0.857
Uniformity	0.690	0.343	1.000	1.000	0.630	0.807
Mean intensity	0.764	0.649	0.867	0.813	0.735	0.834
Standard deviation	0.539	0.030	0.993	0.800	0.534	0.510
Kurtosis	0.525	0.000	0.993	0.000	0.527	0.565
Skewness	0.782	0.649	0.900	0.853	0.742	0.828

To reduce the photon noise, filters were used in the pre-processing step. As shown in Table 5, multi-feature subsets achieved better classification results than single feature. And the features obtained by filtered images achieved higher ACC and AUC than those without filters (ACC = 0.859, AUC = 0.942). For the three filters, median (ACC = 0.873 and AUC = 0.949) and wiener (ACC = 0.877 and AUC = 0.948) filters have better performance than average filter (ACC = 0.866 and AUC = 0.943) in this study. The average filter may remove some texture information in the thyroid images when the photon noise was filtered out. The classification using feature subset A6, M6 and W6 outperforms the others in this test. The subset of A6, M6 and W6 includes the features obtained by all three filters, which slightly increases the computation burden. However, it reaches high sensitivity and AUC. It is very important for radiologists to minimize the risk of missing nodules that may pose a cancer threat to the patients.

**Table 5:** The classification results of feature subsets without feature selection

Feature subsets	ACC	SEN	SPC	PPV	NPV	AUC
Non-filter	0.859	0.784	0.927	0.905	0.827	0.942
W6	0.877	0.799	0.947	0.930	0.840	0.948
A6, W6	0.873	0.821	0.920	0.902	0.852	0.951
A6, M6, W6	*0.873*	*0.821*	*0.920*	*0.902*	*0.852*	*0.950*
A6, M6, W6, non-filter	0.870	0.821	0.913	0.894	0.851	0.950

A6, M6, and W6 indicate six features with average, median, and wiener filters respectively

**Fig. 4:**
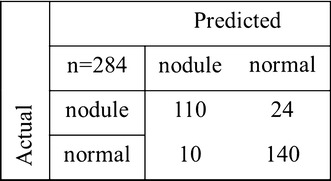
Confusion matrix of the optimal performance of SVM. Thyroid nodules (n = 110) and normal tissues (n = 140) were identified correctly from the samples (n = 284)

In this test, sequential forward feature selection (SFFS) was applied to remove the redundant features and improve the performance of classification. The results of SVM classification with feature selection were shown in Table 6. The confusion matrix of the optimal performance was given in Fig. 4. Entropy and skewness were selected in all the optimized feature subsets. Both features carried much information about the spatial heterogeneity in thyroid tissue, which could be good indicators of thyroid nodules. Using feature selection, the optimal accuracy (0.880), sensitivity (0.821), and AUC (0.953) were obtained in group A6 + M6 + W6. The performance was better than those without using feature selection. It is worth noting that the sensitivity was improved to 0.821, the highest value among these feature subsets. In general, it is important for CAD system to achieve high sensitivity. Because low sensitivity might misdiagnose patients with nodules as healthy ones, which may lead to delay treatment, or even no treatments.

**Table 6:** The results of SVM classification with feature selection

Feature subsets	Selected features	ACC	SEN	SPC	PPV	NPV	AUC
W6	e1, u1, m1, sd1, k1, s1	0.877	0.799	0.947	0.930	0.840	0.948
M6, W6	e1, u1, sd1, k1, s1, e2, u2, m2, k2, s2	0.880	0.813	0.940	0.924	0.849	0.950
A6, M6, W6	k1, s1, m2, k2, s2, e3, u3, sd3, s3	*0.880*	*0.821*	*0.933*	*0.917*	*0.854*	*0.953*
A6, M6, W6, non-filter	sd1, m2, sd2, k2, s2, e4, s4	0.877	0.813	0.933	0.916	0.849	0.950

W6, M6, A6, and non-filter indicate the six features with wiener (group 1), median (group 2), average intensity (group3), and without filter (group 4) respectively. In feature selected column, for example, e1 means entropy in group 1 and k3 means the kurtosis in group 3

To evaluate the performance of classifier of SVM, back propagation artificial neural network (BP-ANN) and linear discriminant analysis (LDA) with leave one out strategy were applied. The BP-ANN model comprised one hidden layer with ten nodes. The output layer included benign and malignant levels. The transfer function of the hidden layer nodes was tansig, and the transfer function of the output layer nodes was purelin. This study applied a classic linear discriminant analysis (LDA). The aim was to find the discriminant function, a parameter that allows for the optimal separation or grouping of data based on their main characteristics. Results of three classifiers are shown in Table 7. SVM has the best performance among three classifiers.

**Table 7:** The results of BP-ANN, LDA, and SVM classification

Classifier	ACC	SEN	SPC	PPV	NPV	AUC
BP-ANN	0.852	0.784	0.913	0.890	0.825	0.918
LDA	0.856	0.791	0.913	0.891	0.830	0.928
SVM	*0.880*	*0.821*	*0.933*	*0.917*	*0.854*	*0.953*

#### Feature assessment

The thyroid gland is a component of the endocrine system. It controls the metabolic process in an organism. The thyroid nodule is a common endocrine disease [26]. The overwhelming majority of thyroid tumours are primary epithelial neoplasms composed of follicular cells [27]. Tumour nodule in the thyroid will make the structure different from normal tissue. Benign nodule grows slowly with capsule and has a clear border against normal tissue. Cells in malignant nodule grow aggressively without obvious borders, and even invade the thyroid capsule. Most of the nodules show low intensity in CT images, because the cell in the nodules cannot absorb the iodine. For example, thyroid cyst represents water-like intensity due to its fluid-filled region. However, some nodules show high intensity if there are calcifications. The nodules cause the change of intensity in CT images (spatial grey heterogeneity) and make the texture feature different from the normal thyroid tissues. So it is possible to discriminate nodules from normal tissues by using the pixel intensity (Figs. 2, 3).

**Fig. 5:**
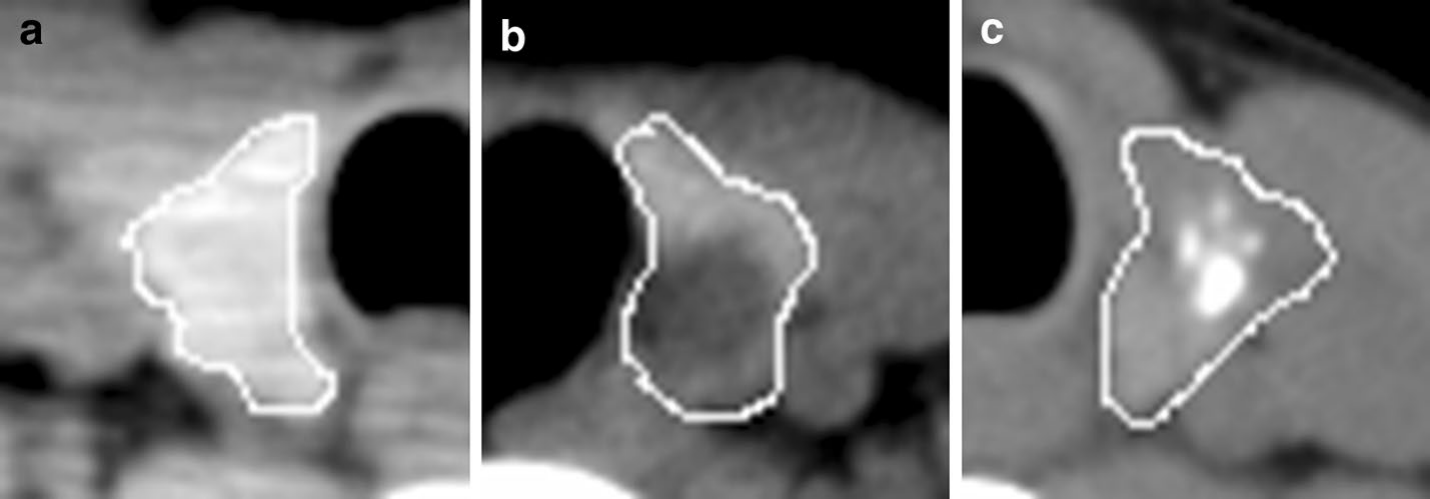
Examples of the normal tissue (**a**) and thyroid nodule (**b**, **c**). The entropy, standard deviation and kurtosis in image a (0.939, 0.021, and 0.001 respectively) are less than those in image** b** and** c** (0.977, 0.029, 0.013 and 0.981, 0.064, 0.006). On the contrary, uniformity in** a** (0.011) is higher than those in** b** (0.002) and** c** (0.004)

The first order texture features could indicate pixel intensity heterogeneity in CT image. The entropy shows the amount of information in ROI. It describes the randomness and irregularity of pixel intensity. Uniformity indicates the distribution of image intensity levels. The presence of cyst and calcification can reduce the uniformity. For mean intensity, compared with normal thyroid tissue (Fig. 5a), it decreases with the existence of cysts (Fig. 5b) and increases with the existence of calcifications (Fig. 5c). So the mean intensity may remain unchanged if both calcification and cyst exist in the same ROI. Standard deviation describes the variation from the mean intensity. The normal tissue has smaller standard deviation than the nodules. The image intensity inside the thyroid is homogeneous since the normal thyroid cells have similar characteristics in function. Kurtosis and skewness indicate the bulging and the asymmetry of the intensity distribution in ROI, respectively.

The gray level co-occurrence matrix (GLCM) was utilized in classification of SVM. The ACC, SEN, SPC, PPV, NPV, and AUC of GLCM are 0.813, 0.710, 0.911, 0.879, 0.775, 0.900 respectively. The first order features have better performance than GLCM. The features of GLCM include angular second moment, correlation degree, entropy, contrast, inverse difference moment, sum average, sum entropy, sum variance, variance, difference average, inertia, difference variance, and difference entropy.

In this preliminary study, we extracted the first order statistic features, and used support vector machine to identify the normal thyroid tissues and nodules based on the CT images. Our method achieved high accuracy (ACC = 0.880, AUC = 0.953). However, there are still some limitations in this research work. (1) High-dimensional image description could be used in the future study, such as wavelet, local binary pattern operator, and etc. (2) Cutting edge techniques in machine learning should be introduced in thyroid CAD system, such as deep neural network, and deep random forest [28]. Deep learning method benefits from massive amounts of labelled data, and give computers the ability to interpret the images. (3) To feed the future CAD system, we need to construct a much bigger dataset. Obtaining high quality annotated datasets remain a costly challenge. The automatic thyroid segmentation in CT images, as part of the pre-processing method, has to be studied further.

## Conclusions

In this study, we presented a CAD system to detect thyroid nodules in CT images. The first order statistic features, including entropy, uniformity, mean intensity, standard deviation, kurtosis and skewness, were calculated to represent the spatial heterogeneity in thyroid images. SVM model was used to identify the normal thyroid tissue and nodule. We further evaluated three filters and different feature subsets to optimize the performance of the classification. The results demonstrated that our method can provide good detection of thyroid nodules. The accuracy, sensitivity, specificity, positive predictive value, and negative predictive value achieve 0.880, 0.821, 0.933, 0.917, 0.854, and 0.953, respectively. The results demonstrated that the first order statistics could be used as imaging biomarkers. The presented CAD system has potential to assist the radiologists to detect the nodules in computed tomography images and release their burden.

## Abbreviations

CT: computed tomography
US: ultrasound
ROIs: regions of interest
SVM: support vector machine
PPV: positive predictive value
NPV: negative predictive value
TNs: incidental thyroid nodules
CAD: computer aided detection
PACS: picture archiving and communication system
PTC: papillary thyroid cancer
FTC: follicular thyroid cancer
AUC: area under the receiver operating characteristic curve
SFFS: sequential forward floating selection
